# Development and Validation of a ^18^F-FDG PET-Based Radiomic Model for Evaluating Hypermetabolic Mediastinal–Hilar Lymph Nodes in Non-Small-Cell Lung Cancer

**DOI:** 10.3389/fonc.2021.710909

**Published:** 2021-09-08

**Authors:** Ming-li Ouyang, Yi-ran Wang, Qing-shan Deng, Ye-fei Zhu, Zhen-hua Zhao, Ling Wang, Liang-xing Wang, Kun Tang

**Affiliations:** ^1^Department of Respiratory Medicine, The First Affiliated Hospital of Wenzhou Medical University, Wenzhou, China; ^2^Department of Medical Engineering, The First Affiliated Hospital of Wenzhou Medical University, Wenzhou, China; ^3^Department of Radiology, The First Affiliated Hospital of Wenzhou Medical University, Wenzhou, China; ^4^Department of Respiratory Medicine, Taizhou Hospital of Zhejiang Province Affiliated to Wenzhou Medical University, Taizhou, China; ^5^Department of Radiology, Shaoxing People’s Hospital, Shaoxing Hospital of Zhejiang University, Shaoxing, China; ^6^Department of Nuclear Medicine, The First Affiliated Hospital of Wenzhou Medical University, Wenzhou, China

**Keywords:** non-small cell lung cancer (NSCLC), hypermetabolic lymph node, metastasis, positron emission tomography/computed tomography (PET/CT), radiomics

## Abstract

**Background:**

Accurate evaluation of lymph node (LN) status is critical for determining the treatment options in patients with non-small cell lung cancer (NSCLC). This study aimed to develop and validate a ^18^F-FDG PET-based radiomic model for the identification of metastatic LNs from the hypermetabolic mediastinal–hilar LNs in NSCLC.

**Methods:**

We retrospectively reviewed 259 patients with hypermetabolic LNs who underwent pretreatment ^18^F-FDG PET/CT and were pathologically confirmed as NSCLC from two centers. Two hundred twenty-eight LNs were allocated to a training cohort (LN = 159) and an internal validation cohort (LN = 69) from one center (7:3 ratio), and 60 LNs were enrolled to an external validation cohort from the other. Radiomic features were extracted from LNs of PET images. A PET radiomics signature was constructed by multivariable logistic regression after using the least absolute shrinkage and selection operator (LASSO) method with 10-fold cross-validation. The PET radiomics signature (model 1) and independent predictors from CT image features and clinical data (model 2) were incorporated into a combined model (model 3). A nomogram was plotted for the complex model, and the performance of the nomogram was assessed by its discrimination, calibration, and clinical usefulness.

**Results:**

The area under the curve (AUC) values of model 1 were 0.820, 0.785, and 0.808 in the training, internal, and external validation cohorts, respectively, showing good diagnostic efficacy for lymph node metastasis (LNM). Furthermore, model 2 was able to discriminate metastatic LNs in the training (AUC 0.780), internal (AUC 0.794), and external validation cohorts (AUC 0.802), respectively. Model 3 showed optimal diagnostic performance among the three cohorts, with an AUC of 0.874, 0.845, and 0.841, respectively. The nomogram based on the model 3 showed good discrimination and calibration.

**Conclusions:**

Our study revealed that PET radiomics signature, especially when integrated with CT imaging features, showed the ability to identify true and false positives of mediastinal–hilar LNM detected by PET/CT in patients with NSCLC, which would help clinicians to make individual treatment decisions.

## Introduction

Lung cancer is still the leading cause of cancer-related mortality worldwide (1). Non-small-cell lung cancer (NSCLC) accounts for about 85% of lung cancers (2). The occurrence of contralateral or multiregional mediastinal–hilar lymph node metastasis (LNM) in NSCLC might exclude the patient from primary surgery (3, 4), which is significantly associated with unfavorable clinical prognosis. Therefore, accurate lymph nodal staging is critical for determining the treatment options in patients with NSCLC for clinicians.

In recent years, ^18^F-fluorodeoxyglucose (^18^F-FDG) positron emission tomography/computed tomography (PET/CT) has played an important role in evaluating lymph node (LN) status, which can provide both anatomic and glucose metabolic information (5). Numerous studies have shown that intrathoracic nodal status is considered to be positive for metastatic spread if the activity of the node was higher than the mediastinal background (6–8). However, a high level of FDG uptake can also be detected in benign LNs, illustrating the risk of misjudging inflammatory and reactive lymph nodes for metastatic lymph nodes (9, 10). The variable positive predictive rate for assessment of LN involvement with PET/CT has also been recognized, ranging from 32.3% to 89% (9–11). Obviously, the false-positive results of patients with NSCLC could misguide clinicians in making treatment decisions, such as missing surgery or more aggressive treatments, and ultimately influence prognosis. Although PET/CT has a good negative predictive value for the investigation of LNM (9), we have done some studies and published several papers (12, 13). Therefore, various practice guidelines recommend invasive staging procedures with potential side effects for patients with PET positive lymph nodes (14).

To exhaust the full potential information of noninvasive radiology images, a more sophisticated approach is urgently needed. Radiomics is a recently emerging technique for high-throughput mining of tumor characteristics from the images, which reflects the underlying tumor biology and behavior (15, 16). In the field of radiomics analysis based on PET/CT, there has been recently increasing evidence demonstrating the advantages of radiomics as a noninvasive approach in the diagnosis, staging, prognosis, and treatment outcome of lung cancer (13, 17–19). Simultaneously, several studies have focused on assessing the lymph node metastasis using the machine learning algorithm in NSCLC. Yin et al. (20) built a support vector machine model to predict the mediastinal LNM. Pak et al. (21) developed a decision tree model to detect the metastatic mediastinal LNs. However, there are currently few PET-based radiomics nomograms for evaluating hypermetabolic mediastinal–hilar LNs of NSCLC. Moreover, traditional methods for discriminating metastatic LNs are based on CT morphological features. Thus, the purpose of our study was to develop a predictive model that combined ^18^F-FDG PET radiomics features and conventional CT image features to identify true and false positives of mediastinal–hilar lymph node metastasis detected by PET/CT in patients with NSCLC and to validate the predictive value of the model in an independent external data set.

## Materials and Methods

### Patient and Lymph Node Selection

In this retrospective study, we screened and collected ^18^F-FDG PET/CT and clinical data about patients as the primary cohort in The First Affiliated Hospital of Wenzhou Medical University from January 2012 to September 2020. The patients for the external validation cohort were selected from Shaoxing People’s Hospital from November 2016 to December 2020. The study was approved by The Institutional Review Boards of the two participating institutions, and informed consent was waived for this retrospective study.

The specific inclusion criteria were as follows: 1) with pathological diagnosis of NSCLC, 2) with systematic hilar and mediastinal lymph node dissection or the positive results of endobronchial ultrasound-guided transbronchial needle aspirate (EBUS-TBNA) biopsy due to the high false-negative rates [Several studies showed that the positive predictive value and negative predictive values of EBUS-TBNA ranged 92%–100% and 11–97%, respectively (22, 23)], and 3) underwent ^18^F-FDG PET/CT examination. The exclusion criteria for patients were 1) the interval between the pathologic findings and PET/CT scan more than 3 weeks, 2) the patient without the increased FDG uptake LNs, 3) with distant metastasis, 4) with history of other cancer, 5) received neoadjuvant chemoradiotherapy, and 6) images with poor quality as follows: 1) the leakage of ^18^F-FDG at the injection site, which leads to a decrease of FDG uptake of images; 2) images with inferior quality due to noise, respiratory artifacts, or other movement artifacts; and 3) missing PET images or CT images. The exclusion criteria for LNs were 1) mediastinal–hilar LN uptake below or comparable to mediastinal background activity, 2) the boundary between the hypermetabolic LNs and the primary lesions not clear on PET images, 3) with a short-axis diameter of LNs less than 5 mm insufficient to outline a valid volume of interest (VOI), and 4) VOIs did not have enough voxels (at least 64 voxels).

Specific process of determining the pathology of LNs was as follows: 1) radiological, surgical, and pathological LN division all followed the eighth edition of the Union for International Cancer Control TNM classification. The histological findings served as the reference standard for comparison with the PET/CT findings (2). When the LNs dissected in the operation or performed by EBUS-TBNA were pathologically proven to be metastatic in one region, then the identified LNs with hypermetabolic activity on PET images in the same region were regarded as metastatic. 3) If all the harvested LNs within surgery were pathologically proven to be non-metastatic in one region, then the identified hypermetabolic LNs on PET images in the same region were considered as non-metastatic. 4) If systematic LN dissection did not include one region, then the hypermetabolic LNs on PET images in this region were excluded. The detailed flowchart of enrollment is shown in **Supplementary Material Image 1**.

**Figure 1:**
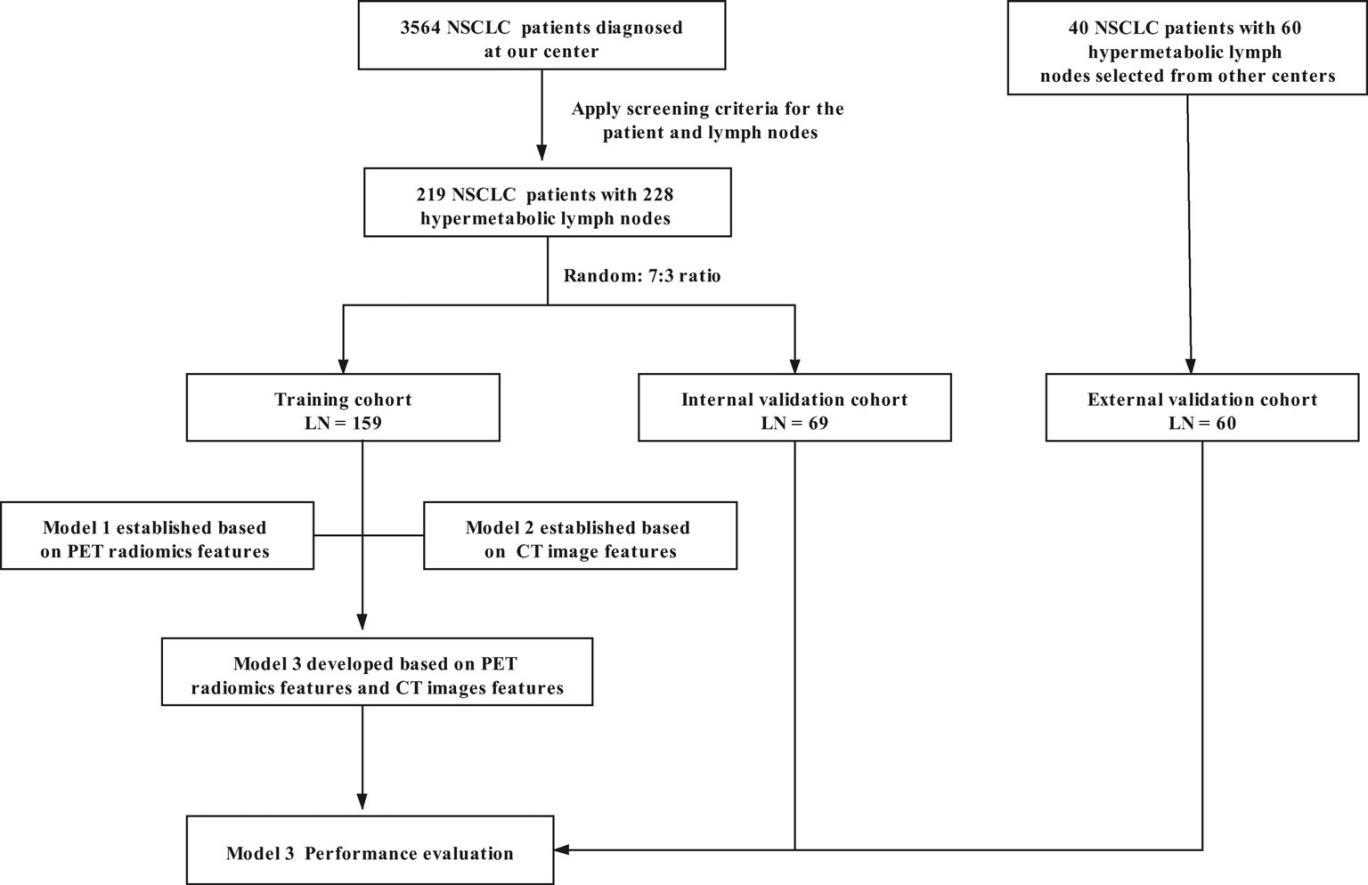
The flowchart shows the process of LN enrollment and scheme.

According to the above criteria, 228 lymph nodes (219 NSCLC patients) were identified as the primary cohort, which were assigned to a training cohort (LN = 159) and an internal validation cohort (LN = 69) by the random split-sample (7:3) method (**Figure 1**). Sixty lymph nodes (40 NSCLC patients) were included in the external validation cohort.

### PET/CT Protocol

^18^F-FDG PET/CT scanning was performed on either Gemini TF 64 (Philips, the Netherlands, 219 patients, 84.6%) from The First Affiliated Hospital of Wenzhou Medical University or General Electric Discovery Elite (Waukesha, WI, 40 patients, 15.4%) from Shaoxing People’s Hospital. All patients were instructed to fast for at least 6 h (blood glucose levels below 110 ml/dl) before being injected with ^18^F-FDG (3.7–5.55 MBq/kg). PET/CT images were acquired from the skull base to the mid-thigh after the injection with an average interval of 59.6 ( ± 7.8) min. Attenuation correction of the PET scan was based on the CT images. The detailed acquisition parameters are given in **Supplementary Material Table 1**, according to the Image Biomarker Standardization Initiative (IBSI) guidelines (24).

### PET Radiomic Feature Extraction

FDG-PET images with the DICOM format were imported into LIFEx freeware (version 6.30, http://www.lifexsoft.org), and the software was used to extract radiomics features. The VOI of the LN was manually delineated and segmented slice by slice using 3D drawing tools by two experienced nuclear medicine physicians who were blinded to pathological results. Forty percent of the maximum standardized uptake value (SUVmax) was adopted as a threshold to optimize the VOI. VOIs were placed to avoid adjacent vessels, bronchi, and the chest wall. For image preprocessing, the resampling voxel size was set at 4 × 4 × 4 mm on PET images in all the patients. We chose an isotropic voxel size of 4 mm because the original voxel size was closer to 4 mm in the two centers of our study. Intensity discretization and rescaling were performed automatically by the software. For PET images, intensity discretization was processed by decreasing the continuous scale to 64 bins, and the intensity rescaling bonds ranged from 0 to 20. Radiomic features were divided into seven groups: conventional features (e.g., SUVmax, SUVmean), discretized/histogram (HISTO), shape, gray-level zone-length matrix (GLZLM), gray-level run-length matrix (GLRLM), neighborhood gray-level different matrix (NGLDM), and gray-level co-occurrence matrix (GLCM). Radiomic features extracted from PET images are shown in **Supplementary Material Table 2**. Source data are available in **Supplementary Material Table 3**.

### CT Image Analysis

Based on the CT component of the PET/CT images, the analysis of the image features was performed by two experienced nuclear medicine physicians. In case of disagreements, consensus was sought by discussion. The following conventional CT features about LN were analyzed: size, shape, margin, calcification, cystic change, CTmax, and CTmean. The size of the lymph node was defined by the maximal short-axis diameter. We defined round or oval as regular shape according to CT images, and any other shape was defined as irregular. CTmax and CTmean were measured at the largest section of LN on transverse CT images.

### Radiomic Feature Screening and Signature Building

To verify interobserver reliability, the intraclass correlation coefficient (ICC) was performed for each VOI-based radiomic feature. Features with ICC greater than 0.8 were selected for subsequent investigation. Additionally, least absolute shrinkage and selection operator (LASSO) regression was applied to further screen the optimal subset from the training cohort and develop a formula by multivariable logistic regression to calculate the radiomics score (Rad-score). Ten-fold cross-validation was used to tune the optimal lambda (λ) value that was the minimum mean cross-validated error.

### Construction of Three Prediction Models

#### Model 1: PET Radiomics Signature

In the training cohort, the optimal PET radiomics features identified by the above LASSO logistic regression procedure using 10-fold cross-validation were utilized to construct a radiomics signature (Rad-score), which formed the prediction model 1.

#### Model 2: CT Image Features and Clinical Data

The differences between LNM and non-LNM groups in CT image features and clinical data were firstly assessed using univariable logistic regression analysis. Features with p < 0.05 in the univariate analysis were included in subsequent multivariable analysis, and model 2 was built in the training cohort using the backward step-down process.

#### Model 3: Combination of Model 1 and Model 2

The complex model (model 3) was the combination of model 1 and model 2 by multivariable logistic regression using backward stepwise selection in the training cohort.

### Validation of the Models and Clinical Utility

The receiver operating characteristic (ROC) curves and the areas under the curve (AUCs) were used to measure discriminative ability of the three different models in the training, internal validation and external validation cohorts, respectively. The complex model was also implemented into a nomogram to provide the clinicians an intuitive and quantitative tool for the predictive probability of LNM in NSCLC patients with the increased FDG uptake LNs. Calibration curves were applied to assess the consistency between the nomogram-predicted and actual probability by the Hosmer–Lemeshow goodness-of-fit test in the three cohorts, respectively. A p-value of the Hosmer–Lemeshow test > 0.05 indicated a good fit. In addition, decision curve analysis (DCA) was adopted to determine the clinical usefulness of the nomogram by evaluating the net benefits at different threshold probabilities. The TRIPOD reporting checklist was appended as additional file: **Supplementary Material Table 4**.

### Statistical Analysis

IBM SPSS (version 25.0) and R software (version 3.6.3) were used for data analysis. Numerical data were expressed as mean ± standard deviation, and categorical data were described as numbers. Numerical data: for comparisons between two groups, unpaired t test was performed when the variances were homogeneous, and a non-parametric test (Mann–Whitney) was performed for non-homogeneous variances. For comparisons among three groups, ANOVA was used when the variances were homogeneous. Categorical data: the chi-square test for the analysis of two-by-two contingency tables was performed, and Fisher’s exact test for the analysis of multiple rows and columns was performed. All radiomics features were normalized using the function “scale” of the R-package “base.” Univariable and multivariable logistic regression analyses were performed. The LASSO regression was carried out by the “glmnet” package. The nomogram and calibration curves were performed with the “rms” package. The “pROC” package was used to analyze ROC curves. Comparison of ROCs was performed by the Delong test. Origin (version 2018) was used to plot the ROC curves. DCA was generated using the “rmda” package. All statistical tests were two-sided, and p value < 0.05 was considered statistical significance.

## Results

### Clinical and Pathological Characteristics

The clinicopathologic characteristics in the training (LN= 159), internal (LN= 69), and external validation cohorts (LN = 60) are summarized in **Table 1**. There were no statistical differences in gender (p =0.789), age (p = 0.09), pathological type (p = 0.333), LN station (p = 0.153), or pathological acquisition of LNs (surgery/TBNA) (p= 0.572) among the three cohorts.

**Table 1:** Demographics and lymph node distribution of training and two validation cohorts.

Characteristics	Training cohort LN = 159	Internal validation cohort LN = 69	External validation cohort LN = 60	p value
**Gender (from M/F patients)**	113/46	47/22	40/20	0.789
**Age (years)**	64.4 ± 9.0	65.2 ± 8.7	67.2 ± 6.9	0.090
**Lung cancer _pathology (ADA/** **SQCC/other types of NSCLC)**	93/49/17	46/17/6	29/22/9	0.333
**LN station**				0.153
**1R/1L/2R/2L/3A/4R/4L**	2/1/4/0/1/25/5	1/1/0/1/0/9/2	2/0/0/0/2/9/3	
**5/6/7/8/9L/10R/10L**	10/5/32/1/1/33/17	1/1/14/0/1/15/9	2/1/8/0/1/9/4	
**11R/11L/12R/12L/13R**	11/11/0/0/0	11/2/0/1/0	8/6/2/1/2	
**Means of obtaining pathology (surgery/TBNA)**	127/32	51/18	48/12	0.572

LN, lymph node; M, man; F, female; ADA, adenocarcinoma; SQCC, squamous-cell carcinoma, NSCLC, non-small cell lung cancer.

### Model 1: PET Radiomics Signature

**Figure 2:**
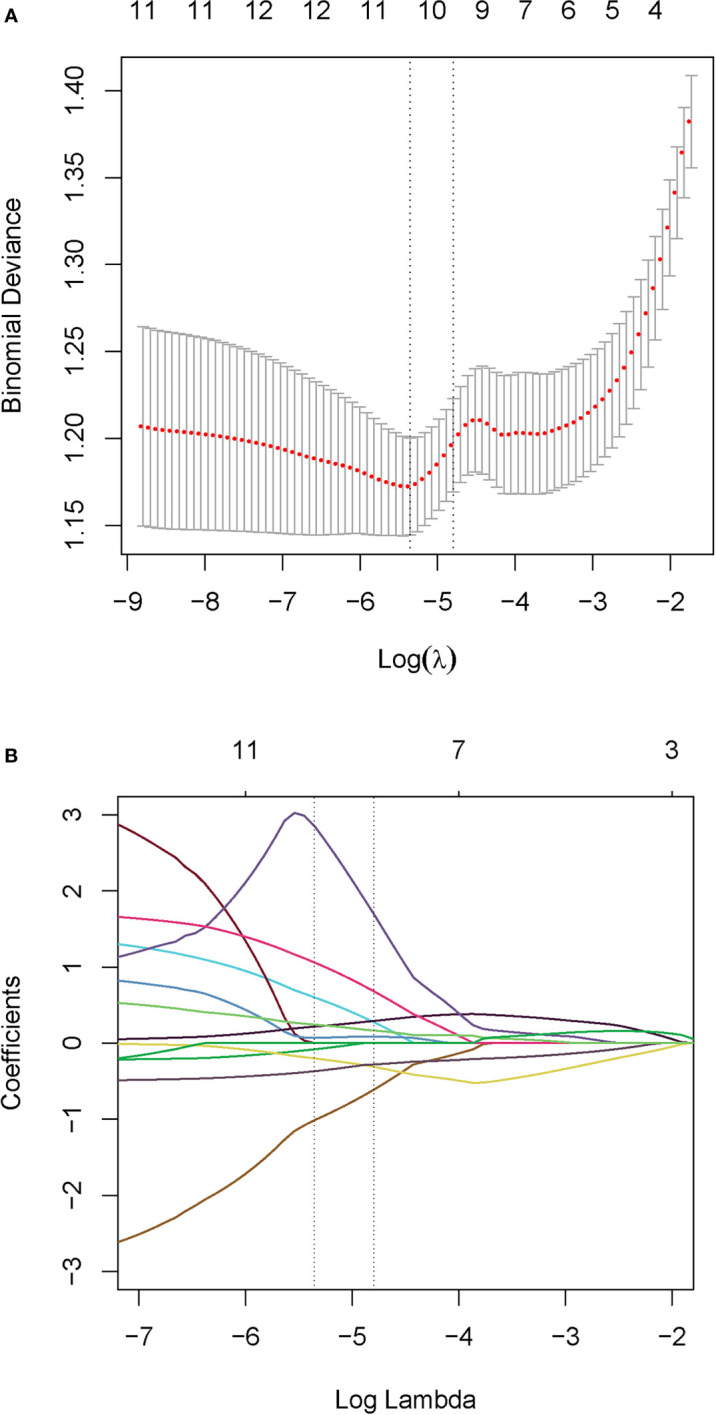
Radiomics feature selection. **(A)** Tuning parameter (λ) selection with 10-fold cross validation in the LASSO model *via* minimum criteria. Optimal feature selection according to AUC value. **(B)** LASSO coefficient profiles of the 70 radiomic features. Dotted vertical lines defined the optimal values of λ. The optimal λ value of 0.00472 with log (λ) of -5.36 resulting in nine nonzero coefficients were selected.

Due to all extracted features with the inter-reader ICC values >0.8 (mean ± standard deviation, 0.865 ± 0.047), all the 70 original radiomic features were selected for subsequent investigation. Then, the contours drawn by a higher senior physician were finally used to calculate the radiomic features for the modelling. Subsequently, 70 radiomic candidate features were reduced to 9 with nonzero coefficients by the LASSO regression (**Figure 2**). Then, by multivariable logistic regression analysis, five features were not statistically significant (p > 0.05). Thus, only four features were used to build the model in the training cohort. The four selected radiomic features were DISCRETIZED_HISTO_Excess Kurtosis, GLRLM_GLNU (Gray-Level Non-Uniformity for run), GLRLM_RLNU (Run Length Non-Uniformity), and NGLDM_ Coarseness. Eventually, the four optimal radiomics features were incorporated into radiomics signature. PET_Rad_score = 1.004 + 1.151 × DISCRETIZED_HISTO_Excess Kurtosis − 1.716 × GLRLM_ GLNU + 5.651 × GLRL M_RLNU +1.760 × NGLDM_ Coarseness (**Table 3**). The AUC of the model 1 was 0.820 in the training cohort. The sensitivity, specificity, and accuracy were 61.80%, 90.00%, and 74.22%, respectively (**Table 4**).

### Model 2: CT Image Features and Clinical Data

A comparison of CT image features and clinical data between LNM and non-LNM groups in the training (LNM = 89; non-LNM = 70), internal (LNM = 39; non-LNM = 30), and external validation cohorts (LNM = 33; non-LNM = 27) is listed in **Table 2**. Univariate analysis demonstrated that there were no statistical differences in age, pathological type, LN station, shape, margin, calcification, and cystic change between the two groups in all three cohorts. Meanwhile, the univariate analysis showed that LN size and CTmean were deemed significantly different between the two groups in all three cohorts. In the training cohort, there also showed a significant statistical difference in CTmax and gender through univariate analyses. Subsequently, gender, LN size, CTmax, and CTmean were finally selected through multivariable logistic regression with a stepwise backward selection in the training cohort. The results showed that gender and CTmax were not statistically significant (p > 0.05). Thus, only LN size and CTmean were significantly independent predictive factors. The formula of Model 2 = −1.989 + 0.288 × Size− 0.31 CTmean (**Table 3**). The AUC of model 2 was 0.780 in the training cohort. The sensitivity, specificity, and accuracy were 79.78%, 68.57%, and 74.84%, respectively (**Table 4**).

**Table 2:** Comparison of CT image features and clinical data between LNM and non-LNM groups in the three cohorts.

Characteristics	Training cohort (LNM = 89; non-LNM = 70)			Internal validation cohort (LNM = 39; non-LNM = 30)			External validation cohort (LNM = 33; non-LNM = 27)		
	LNM	non-LNM	P	LNM	non-LNM	P	LNM	non-LNM	P
Gender (from M/F patients)	57/32	56/14	0.029	27/12	20/10	0.821	26/7	14/13	0.031
Age (years)	63.4 ± 9.6	65.7 ± 8.0	0.105	63.7 ± 9.7	67.1 ± 6.8	0.111	66.5 ± 6.4	68.2 ± 7.5	0.350
Lung cancer_pathology			0.407			0.182			0.720
(ADA/SQCC/Other types of NSCLC)	51/26/12	42/23/5		25/8/6	21/9/0		17/11/5	12/11/4	
LN station			0.085			0.117			0.455
1R/1L/2R/2L/3A/4R/4L	2/1/4/0/1/14/2	0/0/0/0/0/11/3		1/1/0/1/0/7/2	0/0/0/0/0/2/0		2/0/0/0/2/5/1	0/0/0/0/0/4/2	
5/6/7/8/9L/10R/10L	7/3/20/1/1/13/7	3/2/12/0/0/20/10		0/1/8/0/1/4/4	1/0/6/0/0/11/5		2/0/5/0/1/2/3	0/1/3/0/0/7/1	
11R/11L/12R/12L/13R	5/8/0/0/0	6/3/0/0/0		7/1/0/1/0	4/1/0/0/0		3/4/1/1/1	5/2/1/0/1	
Size (mm)	13.1± 3.6	10.7 ± 2.7	< 0.001	13.2 ± 3.5	10.7 ± 1.8	0.003	11.6 ± 3.0	9.8 ± 2.1	0.019
Shape			0.445			0.093			0.136
Regular	78(87.6)	64 (91.4)		32 (82.1)	29 (96.7)		32 (97.0)	23 (85.2)	
Irregular	11(12.4)	6 (8.6)		7 (17.9)	1 (3.3)		1 (3)	4 (14.8)	
Margin			0.095			0.093			0.648
Clear	73(82.0)	64 (91.4)		32 (82.1)	29 (96.7)		28 (84.8)	24 (88.9)	
Unclear	16(18.0)	6 (8.6)		7 (17.9)	1 (3.3)		5 (15.2)	3 (11.1)	
Calcification			0.197			0.151			0.302
None	83 (93.3)	61 (87.1)		35 (89.7)	23 (76.7)		29 (87.9)	21 (77.8)	
Presence	6 (6.7)	9 (12.9)		4 (10.3)	7 (23.3)		4 (12.1)	6 (22.2)	
Cystic change			0.999			0.999			1.000
None	87 (97.8)	70 (100.0)		36 (92.3)	30 (100.0)		32 (97.0)	27 (100.0)	
Presence	2 (2.2)	0 (0.0)		3 (7.7)	0 (0.0)		1 (3.0)	0 (0.0)	
CTmax	50.5 ± 41.6	69.0 ± 48.4	0.021	56.2 ± 53.4	88.4 ± 97.1	0.117	71.6 ± 41.1	97.2 ± 56.1	0.065
CTmean	32.1 ± 21.8	42.9 ± 15.1	0.001	32.2 ± 18.8	54.4 ± 43.4	0.019	40.8 ± 7.3	53.3 ± 16.2	0.001

p value is obtained by using the univariate analysis between each variable and node status.

LNM, lymph node metastasis; non-LNM, no lymph node metastasis.

**Table 3:** Results of multivariable logistic regression analysis in the three models.

Models	Included features	Odds ratio (95% CI)	p value	Coefficient	Intercept
Model 1	Radiomics signature				**1.004**
	DISCRETIZED_HISTO_ExcessKurtosis	3.160 (1.477–7.228)	0.004	1.151	
	GLRLM_GLNU	0.180 (0.073–0.395)	<0.001	-1.716	
	GLRLM_RLNU	284.479 (26.676–4552.497)	<0.001	5.651	
	NGLDM_Coarseness	5.813 (2.453–15.202)	<0.001	1.760	
Model 2	Conventional CT images and clinical data				**-1.989**
	Size(mm)	1.333 (1.160–1.533)	<0.001	0.288	
	CTmean	0.969 (0.949–0.990)	0.003	-0.31	
Model 3:	Model 1 + Model 2				**0.6949**
	PET_Rad_score	3.073 (2.018–4.679)	<0.001	1.1227	
	Size (mm)	1.118 (0.955–1.309)	0.1659	0.1114	
	CTmean	0.948 (0.926–0.971)	<0.001	-0.0531	

PET_Rad_score, PET radiomics signature; HISTO, histogram; GLRLM, gray-level run-length matrix; GLNU, gray-level non-uniformity for run; RLNU, run length non-uniformity, NGLDM, neighborhood gray-level different matrix; CI, confidence interval.

The bold values refer to the intercepts for calculation formula.

**Table 4:** Performance evaluation of three models in three cohorts.

Model	Training cohort				Internal validation cohort				External validation cohort			
	AUC (95% CI)	Sensitivity (%)	Specificity (%)	Accuracy (%)	AUC (95% CI)	Sensitivity (%)	Specificity (%)	Accuracy (%)	AUC (95% CI)	Sensitivity (%)	Specificity (%)	Accuracy (%)
Model1	0.820 (0.754–0.885)	61.80%	90.00%	74.22%	0.785 (0.663–0.906)	66.67%	86.67%	75.37%	0.808 (0.697–0.919)	66.67%	85.19%	75.00%
Model2	0.780 (0.707–0.853)	79.78%	68.57%	74.84%	0.794 (0.687–0.902)	66.67%	83.33%	73.91%	0.802 (0.690–0.915)	72.73%	81.48%	76.67%
Model3	0.874 (0.821–0.927)	64.05%	94.29%	77.36%	0.845 (0.744–0.946)	61.54%	100%	78.26%	0.841 (0.736–0.945)	87.88%	77.78%	83.34%

AUC, area under the curve; CI, confidence interval.

### Model 3: Combination of Model 1 and Model 2

**Figure 3:**
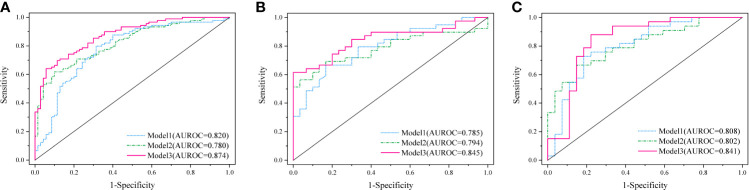
Diagnostic performance of the three established models was evaluated using ROC curves in the training cohort **(A)**, internal **(B)**, and external validation cohort **(C)**.

A complex model (model 3) incorporating independent predictors from model 1 and model 2 was developed by multivariable logistic regression analysis (**Table 3**). Calculation formula = 0.6949 + 1.1227 × PET_Rad_score + 0.1114× Size − 0.0531× CTmean. The difference of LN size in Model 2 was statistically significant (p < 0.001; **Table 3**). However, there was no significant statistical difference in the complex model (p = 0.1659; **Table 3**). The reason for this phenomenon is that there was a low–moderate correlation between LN size and Rad-score (Pearson correlation coefficient = 0.556, p < 0.001). The discriminatory ability of model 3 displayed the highest with an AUC of 0.874 among the three models in the training cohort (sensitivity 64.05%, specificity 94.29%, and accuracy 77.36%) (**Figure 3A** and **Table 4**). Moreover, model 3 was statistically significantly better than both model 1 (p = 0.009) and model 2 (p = 0.011) in the training cohort by the Delong test.

### Validation of the Models and Clinical Utility

Based on the formula of the above three models from the training cohort, the AUC values demonstrated that predictive efficacy was calculated in the internal and external validation cohort. The AUC values were 0.785 (model 1), 0.794 (model 2), and 0.845(model 3) in the internal validation cohort (**Figure 3B**). The AUC values were 0.808 (model 1), 0.802 (model 2), and 0.841 (model 3) in the external validation cohort (**Figure 3C**). The sensitivity, specificity, and accuracy are shown in **Table 4**. Obviously, the complex model showed optimal diagnostic performance among the three cohorts.

**Figure 4:**
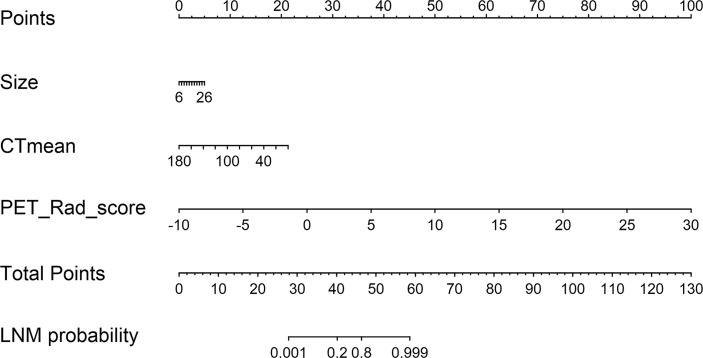
A radiomics nomogram was developed incorporating PET Rad_score with conventional CT images (size and CTmean) in the training cohort.

**Figure 5:**
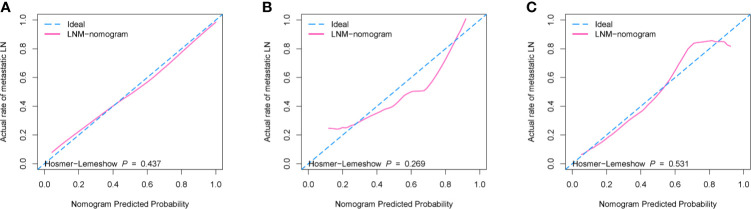
The calibration curves with Hosmer–Lemeshow test of the nomogram (model 3). **(A)** Training cohort. **(B)** Internal validation cohort. **(C)** External validation cohort. The x-axis represents the predicted LNM risk, and the y-axis represents the actual probability of LNM. The closer the diagonal dotted blue line fit is to the ideal line (the pink solid line), the better the predictive ability of the nomogram is.

**Figure 6:**
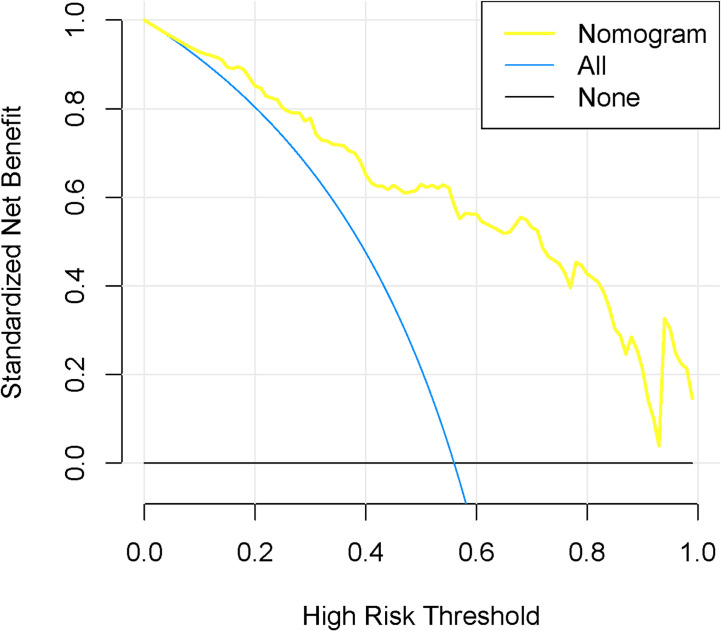
Decision curve analysis for the nomogram in the training cohort. The y-axis represented the net benefit. The blue line represents the assumption that all have LNM. The black line assumes no LNM. The decision curves indicated that the nomogram was clinically useful.

According to the good diagnostic efficiency of the complex model, a nomogram was generated from the training cohort (**Figure 4**). The calibration curves demonstrated good consistency between the nomogram-predicted probability of LNM and the actual LNM rate in the three cohorts (**Figure 5**). There was no statistical significance by the Hosmer–Lemeshow test (p = 0.437, 0.269, and 0.531, respectively), which showed good fits for the predicted and actual probabilities of LNM in the three cohorts (**Figure 5**). Finally, the DCA of the nomogram for the training cohort is presented in **Figure 6**. The decision curve demonstrated that if the threshold probability is more than 10%, using the nomogram to predict LNM adds more benefit than either a “treat all” strategy or a “treat none” strategy.

## Discussion

With respect to PET/CT, the mediastinal–hilar lymph node which had increased glucose uptake is suspected of having malignant infiltration in patients with NSCLC (7). However, false positivity was frequently detected due to the reactive hyperplasia or granulomatous inflammation (9). In this study, we developed and validated a radiomics nomogram that incorporates PET radiomics signature and two CT image features for the identification of metastatic LNs from the increased FDG uptake LNs in patients with NSCLC.

In recent years, growing evidence suggests that radiomics analysis can better reflect the spatial distribution of voxels and enable profiling of intratumoral heterogeneity in a noninvasive way (15, 25). Radiomics data include first-, second-, and higher-order statistics. Scaling correction was applied to the radiomics values in our study. However, this may not take into account site-by-site differences in scanners, i.e., the radiomics batch effect. Approaches such as the ComBat function are available to address such difficulties with radiomics in follow-up studies. The present study applied radiomics analysis of PET and found that metastatic LNs showed significant differences on DISCRETIZED _HISTO_ExcessKurtosis, GLRLM_GLNU, GLRLM_RLNU, and NGLDM_Coarseness. Among the four texture features, DISCRETIZED _HISTO_ExcessKurtosis is the only feature related to the histogram that represents the distribution of gray level within the VOI. This feature is a relatively simple first-order parameter that describes the heterogeneity of lesions (26). Hu et al. (17) found that histogram features (HISTO_Skewness and HISTO_Kurtosis) extracted from PET had a significant value in differentiating solitary lung adenocarcinoma from tuberculosis. GLRLM_GLNU, GLRLM_RLNU, and NGLDM_ Coarseness are the higher-order radiomic features. Several studies have demonstrated that the GLRLM and NGLDM texture analysis can assess the heterogeneity of the lesion (27, 28). Wu et al. (29) reported that the GLRLM textural feature could be useful in differentiating the invasiveness of lung adenocarcinoma. The study by Hoshino et al. (28) presented that NGLDM_Coarseness was correlated with the real miR-1246 expression in the serum of esophageal cancer patients. In the present research, GLRLM_GLNU, GLRLM_RLNU, and NGLDM_Coarseness were obviously different between true and false positives of LNM groups, which indicated that metastatic LNs showed high tissue heterogeneity than non-metastatic LNs in patients with NSCLC. Finally, the AUC values of the PET radiomic model were 0.820, 0.785, and 0.808 in the training, internal, and external validation cohorts, respectively, showing good diagnostic efficacy for LNM.

According to previous studies, combining PET glucose metabolic information and CT morphological features is more accurate than PET or CT alone in predicting LN status (30, 31). Thus, the conventional CT imaging features should be considered to further enhance the diagnostic accuracy. Our study demonstrated that there were statistically significant differences in LN size and CTmean between the LNM group and non-LNM group, while there were no statistical differences in shape, margin, calcification, cystic change, and CTmax. However, Zhao et al. showed that shape, margin, calcification, and cystic change had a significant effect on the risk of LNM in patients with thyroid cancer (32). We thought that the discrepancy might be related to different primary lesions, and more importantly the observer’s subjectivity and poor stability of morphological assessment. For the LN size, numerous studies have proved that it is an important parameter in predicting nodal involvement with malignancy, albeit as a relatively unreliable parameter in the assessment of LNs with small lesions (33, 34). Our results also provide evidence that the LN size was a significantly independent predictive factor in the prediction of LNM. For the density of LNs, several studies have shown that the density of benign LNs tends to have higher attenuation, as a result of the chronic granulomatous inflammation (30, 35), which were consistent with our results. For clinical data, our study showed that all of them did not contribute to the model. The possible reason is that a relatively small number of clinical features were included.

The combined model (model 3) was statistically significantly better than both model 1 and model 2 by the Delong test. Obviously, model 3 showed optimal diagnostic performance for predicting the risk of LNM from the increased FDG uptake LNs in NSCLC among the three cohorts, with an AUC of 0.874, 0.845, and 0.841, respectively. LN size in Model 3 had no statistically significant difference, while a statistically significant difference in RAD-score was observed. The results indicated that RAD-score contributed more to the model than size, and the nomogram based on model 3 also proved this. Furthermore, the nomogram demonstrated satisfactory discrimination and calibration in this internal and external validation study, which could be easily available to generate an individual probability of LNM before treatment. Therefore, the nomogram, which can provide a wealth of complementary information of the images, may further extend our knowledge and improve the diagnosis. Although it still could not replace pathological examination at present, the nomogram could help guide sampling of lymph nodes to increase the positive biopsy rates.

Our study still had some limitations. First, this is a retrospective study, thereby having all of the inherent biases of a retrospective study. Second, there was a relatively small sample size and potential selection bias. The main reason is that a definite pathologic result of each hypermetabolic lymph node was necessary, so the lymph node without the result of pathology was excluded. In addition, we have tried our best to match the precise locations of LNs between PET/CT images and surgical resection or EBUS-TBNA and achieve a one-to-one correspondence. Furthermore, most patients who underwent surgery are early stage with metastasis of only a few or even isolated lymph nodes in our study. Around 9% of patients were excluded due to poor image quality, and we do not know how the results will be thrown by the exclusion of qualitatively judged “bad images”. (This will be improved in the future in clinical routine as follows: 1) intravenous catheters were inserted in cubital veins before injection, and a cotton swab is used to avoid bleeding and leakage from the injection site after injection. 2) The patients should be asked to minimize body movements to avoid movement artifacts, and a relatively low dose of sedative drug would be injected if necessary. In addition, the patients should be asked to reduce the flow of respiratory to minimize breathing motion artifacts. 3) We should strengthen the management of image data transfer and storage.) Third, although we have tried our best to match the precise locations of LNs between PET/CT images and surgical resection, it is difficult to completely avoid the matching bias. A further prospective study is therefore needed to solve this problem. Fourth, for VOI, we did not investigate the sensitivity to observer delineation in addition to laborious and time-consuming manual delineation. Fifth, only the texture features of PET images in this study were extracted because hypermetabolic LNs were selected as subjects. Sixth, our study included a relatively small number of clinical parameters in the predictive model. Seventh, selection of hypermetabolic LNs means this diagnostic performance might disappear in a cohort with a moderately active uptake in LNs. Therefore, prospective multicenter studies with large-scale should be carried out to optimize the robustness and reproducibility of the radiomic model.

## Conclusions

In conclusion, our study revealed that the identified PET radiomics signature has the potential to be used as a biomarker for the identification of LNM from the hypermetabolic LNs of NSCLC. Furthermore, our results provided evidence that a radiomics nomogram that included both the PET radiomics signature and CT images features had a more significant value in identifying true and false positives of mediastinal–hilar LNM detected by PET/CT in patients with NSCLC, which can be used to help clinicians make individual treatment decisions.

## Data Availability Statement

The original contributions presented in the study are included in the article/**Supplementary Material**. Further inquiries can be directed to the corresponding authors.

## Ethics Statement

The studies involving human participants were reviewed and approved by ethics committee of the First Affiliated Hospital of Wenzhou Medical University and Shaoxing People’s Hospital. Written informed consent for participation was not required for this study in accordance with the national legislation and the institutional requirements.

## Author Contributions

KT, L-xW, and M-lO contributed to conception and design of the study. Y-rW, Z-hZ, and M-lO collected the clinical information and the imaging data. Q-SD, Y-fZ and Y-rW performed the VOI segmentation. M-lO, KT and LW analyzed the data. M-lO contributed to the drafting and writing of the manuscript. All authors contributed to the article and approved the submitted version.

## Funding

This work was supported by Zhejiang Public Welfare Technology Application Research Project, China (LGF21H010009).

## Conflict of Interest

The authors declare that the research was conducted in the absence of any commercial or financial relationships that could be construed as a potential conflict of interest.

## Publisher’s Note

All claims expressed in this article are solely those of the authors and do not necessarily represent those of their affiliated organizations, or those of the publisher, the editors and the reviewers. Any product that may be evaluated in this article, or claim that may be made by its manufacturer, is not guaranteed or endorsed by the publisher.
