# Supplementary material for: Development and Validation of a 18F-FDG PET-Based Radiomic Model for Evaluating Hypermetabolic Mediastinal–Hilar Lymph Nodes in Non-Small-Cell Lung Cancer
Source: Front Oncol. 2021 Sep 8;11:710909. doi: 10.3389/fonc.2021.710909 (PMC8457532; doi:10.3389/fonc.2021.710909)
Supplement: Supplementary file 2 [file Table_1.docx]

**Image acquisition parameters.**

| Acquisition  parameters | **GEMINI TF 64** | | **GE Discovery 710** | |
| --- | --- | --- | --- | --- |
|  | PET | CT | PET | CT |
| 18F-FDG activity  (MBq)* | **350** | **——** | **350-550** | **——** |
| Min/bed position | **1.5** | **——** | **2.5** | **——** |
| Crystal | **LYSO** | **——** | **LBS** | **——** |
| Matrix (pixels) | **144×144** | **512×512** | **256×256** | **512×512** |
| Spatial resolution (mm) | **4.9mm** | **0.5mm** | **5.4mm** | **0.5mm** |
| Slice thickness (mm) | **5 mm** | **5 mm** | **5 mm** | **5 mm** |
| Voxel size | **4🞨4🞨4 mm** | **——** | **3.27🞨3.65🞨3.65 mm** | **——** |
| Reconstruction | **OSEM** | **——** | **VUE Point FX** | **——** |
| Slices | **——** | 64 | **——** | **64** |
| Voltage (kV) | **——** | **120 kV** | **——** | **120KV** |
| Tube current (mA) | **——** | **249 mA** | **——** | **80-180 mA** |
| **Detector collimation** | **——** | **64 mm × 0.625 mm** | **——** | **——** |
| **pitch** | **——** | **0.829** | **——** | **——** |
| ***Administered activity was calculated according to the European Association of Nuclear Medicine (EANM) guidelines, version 1.0, and from February 2015, version 2.0 25** | | | | |
