# Supplementary material for: Development and Validation of a 18F-FDG PET-Based Radiomic Model for Evaluating Hypermetabolic Mediastinal–Hilar Lymph Nodes in Non-Small-Cell Lung Cancer
Source: Front Oncol. 2021 Sep 8;11:710909. doi: 10.3389/fonc.2021.710909 (PMC8457532; doi:10.3389/fonc.2021.710909)
Supplement: Supplementary file 3 [file Table_2.docx]

**Texture features calculated from the PET images**

| Feature set | **Conventional features**  CONVENTIONAL_SUVbwmin  CONVENTIONAL_SUVbwmean  CONVENTIONAL_SUVbwstd  CONVENTIONAL_SUVbwmax  CONVENTIONAL_SUVbwQ1  CONVENTIONAL_SUVbwQ2  CONVENTIONAL_SUVbwQ3  CONVENTIONAL_SUVbwSkewness  CONVENTIONAL_SUVbwKurtosis  CONVENTIONAL_SUVbwExcessKurtosis  CONVENTIONAL_SUVbwpeakSphere0.5mL.value.only.for.PET.or.NM.  CONVENTIONAL_SUVbwpeakSphere1mL.value.only.for.PET.or.NM.  CONVENTIONAL_SUVbwcalciumAgatstonScore.onlyForCT.  CONVENTIONAL_TLG.mL..onlyForPETorNM.  **Discretized -derived features**  DISCRETIZED_SUVbwmin  DISCRETIZED_SUVbwmean  DISCRETIZED_SUVbwstd  DISCRETIZED_SUVbwmax  DISCRETIZED_SUVbwQ1  DISCRETIZED_SUVbwQ2  DISCRETIZED_SUVbwQ3  DISCRETIZED_SUVbwSkewness  DISCRETIZED_SUVbwKurtosis  DISCRETIZED_SUVbwExcessKurtosis  DISCRETIZED_SUVbwpeakSphere0.5mL.value.only.for.PET.or.NM.  DISCRETIZED_SUVbwpeakSphere1mL.value.only.for.PET.or.NM.  DISCRETIZED_TLG.mL..onlyForPETorNM.  DISCRETIZED_HISTO_Skewness  DISCRETIZED_HISTO_Kurtosis  DISCRETIZED_HISTO_ExcessKurtosis  DISCRETIZED_HISTO_Entropy_log10  DISCRETIZED_HISTO_Entropy_log2  DISCRETIZED_HISTO_Energy..Uniformity.  **Shape-derived features**  SHAPE_Volume.mL.  SHAPE_Volume.vx.  SHAPE_Sphericity.onlyFor3DROI..  SHAPE_Surface.mm2..onlyFor3DROI.  SHAPE_Compacity.onlyFor3DROI.  **Grey-level co-occurrence matrix (GLCM)**  GLCM_Homogeneity..InverseDifference.  GLCM_Energy..AngularSecondMoment.  GLCM_Contrast..Variance.  GLCM_Correlation  GLCM_Entropy_log10  GLCM_Entropy_log2..JointEntropy.  GLCM_Dissimilarity  **Neighbourhood grey-level different matrix (NGLDM)**  NGLDM_Coarseness  NGLDM_Contrast  NGLDM_Busyness  **Grey-level run-length matrix (GLRLM)**  GLRLM_SRE (Short-Run Emphasis) GLRLM_LRE (Long-Run Emphasis) GLRLM_LGRE (Low Gray-level Run Emphasis ) GLRLM_HGRE (High Gray-level Run Emphasis) GLRLM_SRLGE (Short-Run Low Gray-level Emphasis)  GLRLM_SRHGE (Short-Run High Gray-level Emphasis) GLRLM_LRLGE (Long-Run Low Gray-level Emphasis) GLRLM_LRHGE (Long-Run High Gray-level Emphasis) GLRLM_GLNU (Gray-Level Non-Uniformity for run) GLRLM_RLNU (Run Length Non-Uniformity) GLRLM_RP (Run Percentage)  **Grey-level zone-length matrix (GLZLM)**  GLZLM_SZE (Short-Zone Emphasis) GLZLM_LZE (Long-Zone Emphasis) GLZLM_LGZE (Low Gray-level Zone Emphasis) GLZLM_HGZE (High Gray-level Zone Emphasis) GLZLM_SZLGE (Short-Zone Low Gray-level Emphasis)  GLZLM_SZHGE (Short-Zone High Gray-level Emphasis) GLZLM_LZLGE (Long-Zone Low Gray-level Emphasis) GLZLM_LZHGE (Long-Zone High Gray-level Emphasis) GLZLM_GLNU (Gray-Level Non-Uniformity for zone) GLZLM_ZLNU (Zone Length Non-Uniformity) GLZLM_ZP (Zone Percentage) |
| --- | --- |
